# Interactions of Acetyl-11-Keto-Beta-Boswellic Acid on Catechol-O-Methyltransferase in the Management of Masticatory Myofascial Pain Syndrome

**DOI:** 10.7759/cureus.68300

**Published:** 2024-08-31

**Authors:** Ramya Suresh, Pradeep Kumar Yadalam, Ramya Ramadoss, Karthikeyan Ramalingam, Arvind Muthukrishnan

**Affiliations:** 1 Oral Biology, Saveetha Dental College and Hospitals, Saveetha Institute of Medical and Technical Sciences, Saveetha University, Chennai, IND; 2 Periodontology, Saveetha Dental College and Hospitals, Saveetha Institute of Medical and Technical Sciences, Saveetha University, Chennai, IND; 3 Oral Pathology and Microbiology, Saveetha Dental College and Hospitals, Saveetha Institute of Medical and Technical Sciences, Saveetha University, Chennai, IND; 4 Oral Medicine and Radiology, Saveetha Dental College and Hospitals, Saveetha Institute of Medical and Technical Sciences, Saveetha University, Chennai, IND

**Keywords:** boswellic acid, akba, molecular docking, frankincense, boswellia, masticatory myofascial pain, myofascial pain disorder

## Abstract

Background: Masticatory myofascial pain syndrome (MMPS) is a soft tissue inflammatory disorder that leads to acute or chronic localized pain and stiffness in the muscles. Catechol-O-methyltransferase (COMT) plays a crucial role in mediating pain perceptions in humans by transferring methyl groups to catecholamines. This process requires adequate S-adenosyl methionine (SAMe). A reduction in SAMe leads to COMT inhibition. *Boswellia serrata* possesses multiple therapeutic benefits and is used for treating chronic pain.

Aim: The study aimed to evaluate the therapeutic potential of acetyl-11-keto-beta-boswellic acid (AKBA) by targeting COMT*.*

Methodology: Molecular docking and dynamic simulations were conducted using Desmond software from Schrödinger LLC, USA, to evaluate the interaction between COMT protein and AKBA ligands. The COMT protein structure was sourced from the Protein Data Bank and preprocessed using optimized potentials for liquid simulations. Molecular docking identified potential binding sites between COMT and AKBA through hydrogen bonding, resulting in a docking score of -6.0 kcal/mol.

Results: The molecular docking revealed a docking score of -6.0 kcal/mol for the interaction between COMT and AKBA. The dynamic simulation demonstrated that the COMT-AKBA complex remained stable within a 3.0 Angstrom range over 60 nanoseconds. These findings indicate stable natural small molecular interactions between COMT and AKBA.

Conclusion: AKBA exhibits potential as a therapeutic agent for MMPS, demonstrating stable interactions with COMT. These findings warrant further in vitro and in vivo analyses to confirm efficacy.

## Introduction

Masticatory myofascial pain syndrome (MMPS) is a prevalent condition characterized by chronic pain and discomfort in the muscles which are responsible for mastication or chewing [1]. This syndrome often results from excessive and prolonged muscle activity, leading to the development of sensitive trigger points within the masticatory muscles [2]. These trigger points can cause referred pain, muscle stiffness, and limited jaw movement, significantly impacting an individual's quality of life [3,4]. MMPS has a lifetime frequency of 25% to 85% in the population, with a proclivity for 25- to 60-year-old women. The pathophysiology of MMPS is complex and multifactorial, involving both peripheral and central mechanisms of pain perception [5,6].

Due to significant interindividual heterogeneity in pain perception and drug response, single nucleotide polymorphisms (SNPs) in multiple genes involved in nociceptive pathways have recently been linked to pain sensitivity in individuals with chronic pain syndromes. Among these, the COMT protein encoded by the COMT gene - a major metabolizing enzyme that destroys catecholamines such as dopamine, adrenaline, and norepinephrine is a major determinant [7]. As a result, it helps to maintain homeostasis in several critical biological processes, including pain perception, mood, and responses to both physical and emotional stimuli. A functional SNP in COMT codon 158 (Val158Met) has been found to regulate pain perception and contribute to pain perception disparities [8]. Excessive methylation is a key factor contributing to elevated catecholamine levels, leading to overstimulation of the frontal lobe. This overstimulation impairs COMT activity, which can result in chronic pain, anxiety, depression, and insomnia [9].

*Boswellia serrata* known as frankincense is a natural extract that has been used in ancient times as a medicine to treat various inflammatory diseases. The resinous portion of the plant contains several active components including boswellic acids such as keto boswellic acids, beta boswellic acids, acetyl-11-keto-beta-boswellic acid (AKBA), etc. These acids possess anti-inflammatory, anti-microbial, immunomodulatory, and cytotoxic properties in which AKBA is the most potent inhibitor of lipooxygenase, an enzyme associated with inflammation. AKBA is one of the most active and potent components of *Boswellia serrata *extract and has been extensively studied for its various therapeutic properties. The AKBA is well known for its anti-inflammatory activity as it can inhibit the production of inflammatory molecules in the body, such as cytokines and leukotrienes, which are involved in the immune response. By reducing inflammation, AKBA may be helpful in treating a variety of conditions, such as rheumatoid arthritis, inflammatory bowel disease, and asthma. It has good analgesic properties, which is helpful in treating conditions such as osteoarthritis and chronic pain syndromes. Biologically active natural chemicals aid in the identification of novel therapeutic targets [10-12].

Molecular docking and molecular dynamics simulations are computational methods employed in structural biology and drug discovery. They offer predictions about the binding affinity and dynamics of molecules, such as proteins and ligands, and provide valuable insights into their interactions. It has multiple applications, including drug discovery, which identifies potential drugs by screening a large database of compounds against specific targets. It facilitates the prediction of their binding affinity, thereby guiding the design and optimization of novel pharmaceuticals [13]. This study aimed to show how to use computer-aided drug design to find a potent molecule for targeting COMT protein and to evaluate the usefulness of novel structures in treating MMPS.

## Materials and methods

Protein and ligand selection

Molecular docking and dynamic simulations of the COMTprotein and AKBA were performed using Desmond software of Schrödinger LLC, USA [13,14]. It is a multi-step procedure that is utilized to estimate the binding mode and affinity of a ligand with a receptor, usually a protein. The initial stage involves preparation, wherein the protein and ligand structures are readied by eliminating water molecules, incorporating hydrogen atoms, and allocating suitable charges. This guarantees that the structures are appropriately formatted for the purpose of conducting docking calculations.

Selective algorithm for binding affinity

Subsequently, a search algorithm is utilized to investigate diverse positions and orientations of the ligand within the binding site of the receptor. The algorithm employs a systematic approach to assess the compatibility of individual poses by utilizing scoring functions that estimate the binding affinity between the receptor and ligand. Scoring functions consider various factors, including but not limited to shape complementarity, electrostatic interactions, hydrogen bonding, and hydrophobic interactions. The aforementioned computations offer a numerical evaluation of the potency of the bond between the protein and ligand. Several docking poses are generated, and those with the most elevated scores are chosen as plausible binding modes. The aforementioned postures depict the anticipated alignments and configurations of the ligand situated within the binding site. Subsequent to the docking process, additional methods for examination and enhancement are implemented to verify and enhance the accuracy of the docking outcomes. The process may entail an assessment of the stability and consistency of the docking poses, as well as the implementation of energy minimization or molecular dynamics simulations to enhance the accuracy of the poses.

Structure retrieval

**Figure 1:**
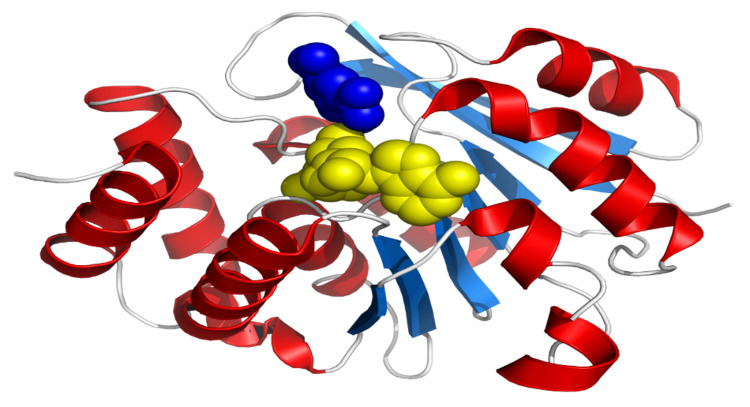
The 3D protein structure of COMT acquired from the PDB database (https://www.rcsb.org/structure/4XUE) COMT: catechol-O-methyltransferase; PDB: protein data bank

The 3D protein structure (Figure 1) of COMT was downloaded from the protein data bank (PDB) database (https://www.rcsb.org/structure/4XUE). The protein structure was processed using the Schrodinger Maestro platform. The Protein Preparation Wizard of Maestro was used to preprocess the ligand-receptor complex. Generally, water molecules in the protein can be easily displaced by the ligand or can cause hindrance for the binding pocket. Therefore, the preprocessing step in protein preparation removes all the hetero atoms and loosely bound water molecules by adding hydrogen ions. The selected protein structure was then examined for gaps built further to fill the loops. After optimization, it was minimized using Optimized Potentials for Liquid Simulations (OPLS) 2005 force field. Conformers for each compound obtained force field estimates by OPLS 2005 between atoms within and between the molecules [15].

The ligand was prepared after downloading the chemical structure of AKBA from pub chem id (https://pubchem.ncbi.nlm.nih.gov/compound/11168203). They were subjected to energy minimization using OPLS 2005 force field to achieve correct bond length, order, and angle with minimal energy. So, this grid-based ligand docking method was used to evaluate the interaction between the protein and the ligand. Docking experiments were the first step in the molecular dynamics simulation of receptor and ligand complexes. In static situations, molecular docking studies predict the ligand-binding states. Docking is beneficial because it gives a static view of a molecule's binding pose at RNA's active site.

Molecular dynamics simulation

The precise depiction of the dynamics and interactions within a bio-molecular system in molecular dynamics simulations necessitates the execution of various crucial procedures. The very first step of the process involves system setup, wherein the fundamental coordinates of the system, encompassing the protein, ligand, and solvent molecules, are established. The assignment of force-field parameters to individual atoms in a given system is a crucial step in characterizing the interatomic interactions within the system. This procedure guarantees that the system is adequately primed for the simulation. The initial stage of the system configuration process is commonly referred to as the equilibration phase. The system undergoes a gradual heating process until it attains the desired temperature, following which it is allowed to equilibrate and attain a state of stability.

In this stage, the biomolecular system undergoes adjustments and interactions with the protein and ligand as the solvent molecules and ions surrounding it undergo changes. The process of equilibration facilitates the attainment of a state that is energetically favorable for the system, thereby ensuring that the ensuing simulation accurately reflects the natural behavior of the system. Upon completion of equilibration, the system proceeds to the production run phase. During this stage, the equations of motion, which are commonly derived from Newton's laws, are solved iteratively over a designated time interval. The process of integrating the equations of motion results in the creation of a trajectory that effectively represents the dynamic behavior of the system throughout a given time period. The simulation facilitates the investigation of molecular movements and interactions by computing the position, velocity, and acceleration of individual atoms. Subsequently, the trajectory that is produced during the production process is subjected to analysis. Diverse analyses may be conducted to derive significant insights from the simulation data. The aforementioned analyses entail scrutinizing the interactions between proteins and ligands to comprehend the manner in which the ligand interacts with the protein. Additionally, the analyses involve exploring the conformational changes of the biomolecule throughout the simulation, approximating binding free energies to assess the potency of the protein-ligand interaction, and investigating the thermodynamic properties of the system. The aforementioned analyses offer valuable insights into the behavior, stability, and energetics of the biomolecular system, thereby facilitating the interpretation of experimental outcomes and the formulation of subsequent experimental designs.

The software used to model molecular dynamics was Desmond from Schrödinger LLC for 100 nanoseconds. By integration of Newton's classical equation of motion, the movements of atoms are stimulated with molecular dynamic stimulation. The status of ligand-binding in the physiological milieu was predicted using molecular dynamic simulations [16].

During the preprocessing phase of the ligand-receptor complex, which included complex optimization and minimization, the Protein Preparation Wizard from Maestro was utilized. To get each system ready, we employed a tool called the System Builder. The default solvent water model TIP3P (three points of transferable intermolecular interaction potential), with an orthorhombic box, was selected to perform the dynamic simulation [17]. Since there was a water-mediated interaction between protein and ligand in this TIPT3 model, water must be included during docking calculation. Two thousand and five force fields were utilized in the simulation. The addition of counterions then neutralized the models. Sodium chloride (NaCl) was added to simulate the physiological condition.

The number of particles, pressure, and temperature (NPT) ensemble with one atmospheric pressure and temperature of 300K standardized the temperature and pressure. This NPT ensemble was maintained throughout the simulation. The model was loosened prior to the dynamic's simulations. In every 100 nanoseconds trajectories were preserved for analysis, and the simulation's stability was confirmed by comparing the root mean square deviation (RMSD) of the protein and ligand over time. The RMSD value is calculated by superimposing the coordinated atoms in the predicted pose onto the experimental pose and then the deviation between the two structures is measured. This calculation involves various steps: First, the subset of atoms involved in the protein-ligand interaction is considered for calculating RMSD. An experimental pose is superimposed onto the structure referred to by aligning the corresponding atoms; this alignment can be performed using various algorithms. The deviation between the corresponding atoms in those two structures is determined by calculation of distance; the squared distances are averaged, and the square root is taken to obtain the RMSD value.

## Results

Molecular docking

**Figure 2:**
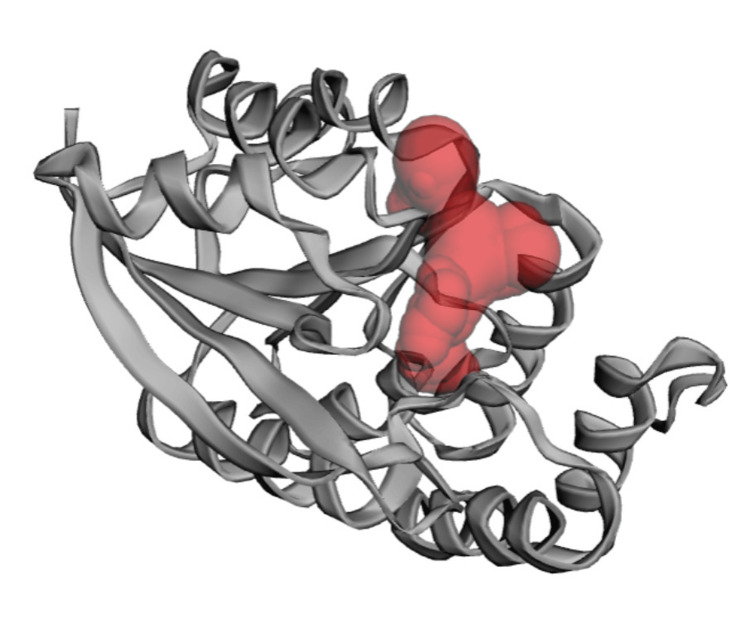
Pocket site of the 3D structure of COMT is centered at coordinates X: 8.111, Y: 11.4591, Z: 15.5386, with a volume encompassing a cubic region of size X: 25, Y: 25, Z: 25. COMT: catechol-O-methyltransferase

Docking offers a valuable static depiction of a molecule's binding pose at the active site of RNA. To delineate the protein's binding site, a grid box was utilized with center coordinates at X: 8.111, Y: 11.4591, Z: 15.5386 and dimensions of X: 25, Y: 25, Z: 25 (Figure 2).

**Figure 3:**
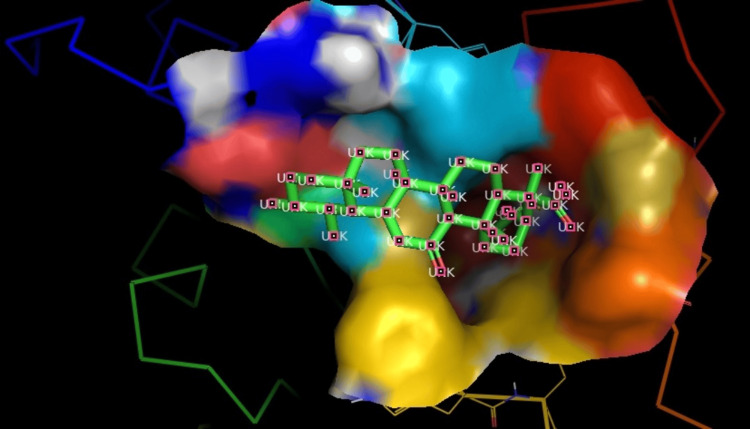
Ligand and protein interaction of COMT and AKBA COMT: catechol-O-methyltransferase; AKBA: acetyl-11-keto-beta-boswellic acid

Hydrogen bonds were identified between the protein (COMT) and the ligand (AKBA) during molecular docking. The molecule docked at -6.0kcal/mol (Figure 3).

**Figure 4:**
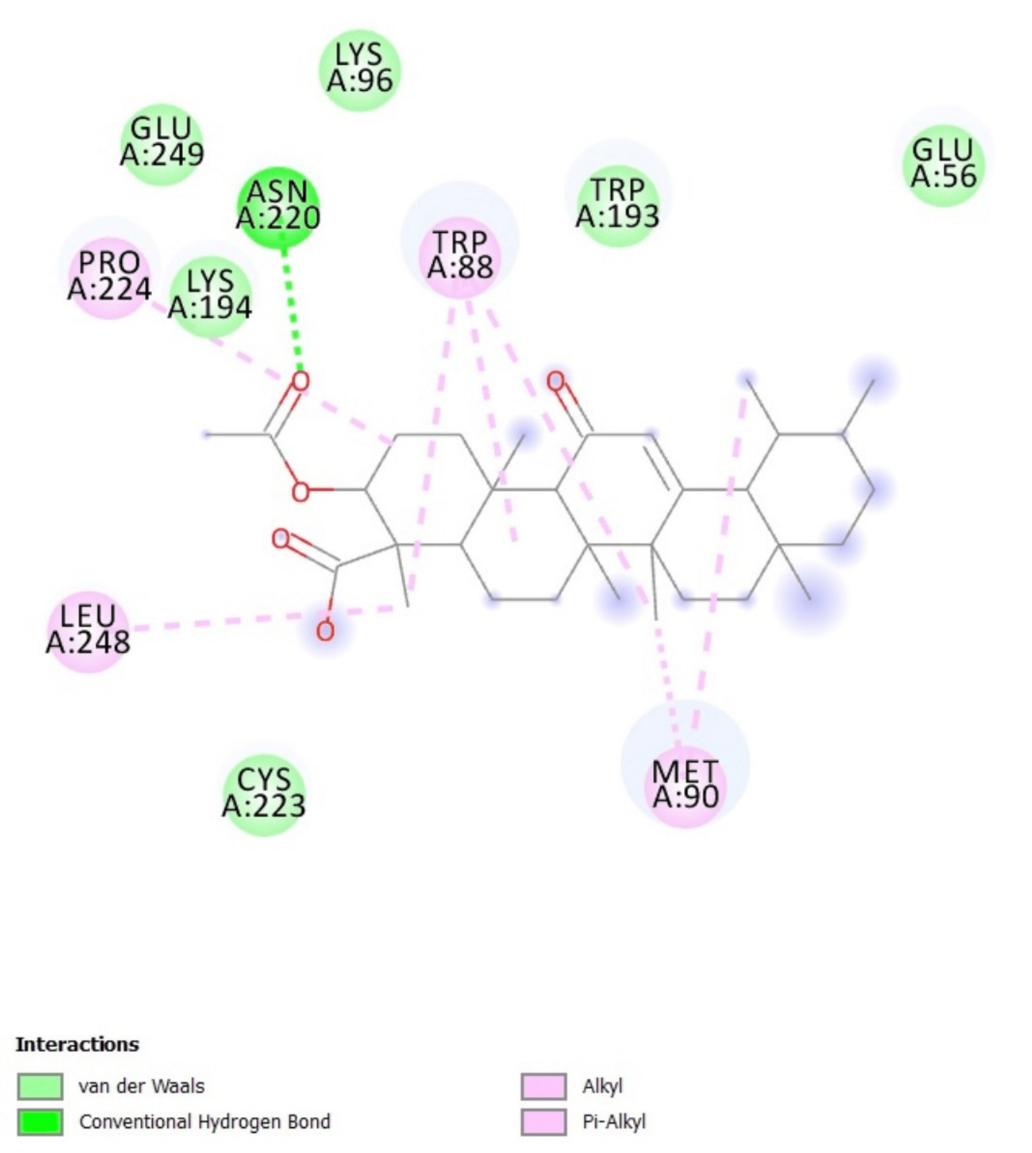
2D interaction showing the binding site residues of COMT and their interactions with the AKBA ligand, including hydrogen bonds and hydrophobic interactions COMT: catechol-O-methyltransferase; AKBA: acetyl-11-keto-beta-boswellic acid; GLU: glutamic acid; LYS: lysine; CYS: cysteine; ASN: asparagine; TRP: tryptophan; MET: methionine; LEU: leucine; PRO: proline

Molecular interaction that highlights the interactions between a molecule and particular amino acid residues within a protein is shown in Figure 4. Van der Waals forces (green), conventional hydrogen bonds (red), and alkyl/Pi-alkyl interactions (pink) are among the interactions. Glutamic acid (GLU) A:249, lysine (LYS) A:194, cysteine (CYS) A:223, asparagine (ASN) A:220, tryptophan (TRP) A:88, methionine (MET) A:90, and leucine (LEU) A:248 are the principal residues.

The amino acids that exhibit the most important interactions are TRP at position 88A, where the distance between ligand atom 2097 and protein atom 401 is 3.62 Å; MET at position 90A, where the distance between ligand atom 2107 and protein atom 414 is 3.81 Å; and LEU at position 248A, where the distance between ligand atom 2097 and protein atom 1899 is 3.84 Å. The crucial sites of contact that stabilize the ligand within the protein's binding site are highlighted by these distances and interactions (Table 1).

**Table 1:** Hydrophobic and hydrogen bond interactions with residues TRP: tryptophan; MET: methionine; LEU: leucine

No.	Residues	Amino acid	Distance	Ligand atom	Protein atom
1	88 A	TRP	3.62	2097	401
2	90 A	MET	3.81	2107	414
3	248 A	LEU	3.84	2097	1899

The interactions between a ligand and protein residues are listed in Table 2. The H-A and D-A distances for residue 220 A (ASN) are 2.29 Å and 2.79 Å, respectively, with a contact angle of 108.67 degrees involving the ligand atom 2106 (O_3_) and the protein atom 1640 (Nam). With an angle of 109.90 degrees, the H-A and D-A distances for residue 223 A (CYS) are 3.60 Å and 4.04 Å, respectively, and they involve the protein atom 2106 (O_3_) and the ligand atom 1665 (O_2_). The hydrogen bonding between the ligand and the protein is described by these values.

**Table 2:** Hydrophobic and hydrogen bond interactions of ASN and CYS ASN: asparagine; CYS: cysteine

No	Residues	Amino Acid	Distance H-A, D-A	Angle (D)	Protein (D)	Side-chain	Atom (D)	Acceptor atom
1	220 A	ASN	2.29, 2.79	108.67	_+_	_+_	1640 (Nam)	2106 (O_3_)
2	223 A	CYS	3.60, 4.04	109.90	-	-	2106 (O3)	1665 (O_2_)

Molecular dynamics simulation

**Figure 5:**
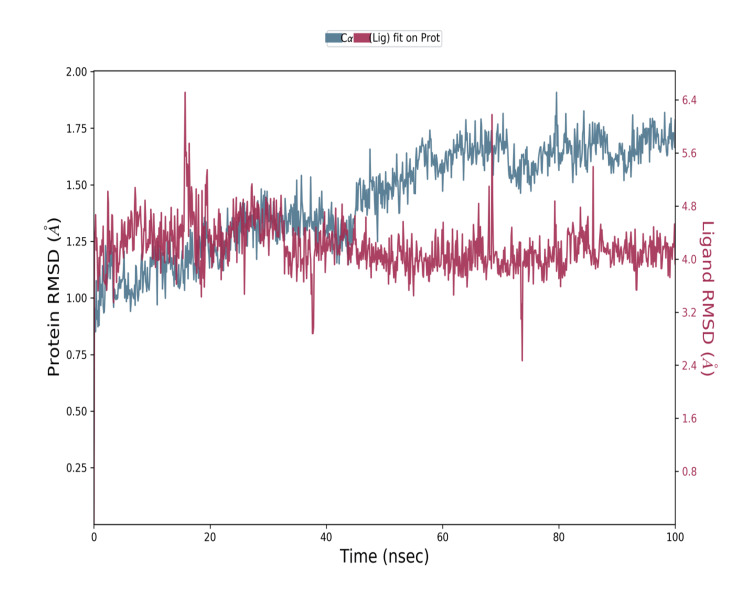
RMSD of the C-alpha atoms for the COMT protein structure with bound AKBA ligand, illustrating the structural deviation between the protein and the ligand RMSD: root mean square deviation; COMT: catechol-O-methyltransferase; AKBA: acetyl-11-keto-beta-boswellic acid

Figure 5 depicts the evolution of RMSD. values for the C-alpha atoms of ligand-bound proteins over time. The proteins in the complex COMT-AKBA (Figure 1). Following that, fluctuations in RMSD values remain stable from 0.8-1.75 to 0-100 nanoseconds for the simulation duration, which is perfectly fine. Ligand RMSD values fluctuations remained stable from 1.0-1.5 Angstrom up to 100 nanoseconds, flipped in ligand mode, regained equilibrium, and remained steady for the simulation duration.

## Discussion

MMPS is a chronic orofacial pain disorder characterized by pain and tenderness in muscles of the jaw and face typically caused by strain, injury, or overuse of masticatory muscles. This condition is commonly associated with malocclusion, stress, and bruxism that increase muscle tension in the jaw area. There are different treatment modalities such as physical therapy, trigger point injections, photobiomodulation therapy, oral appliances, and ultrasound therapies [18].

The therapeutic effects of *Boswellia serrata *extract are attributed to AKBAs, which are the primary active constituents and AKBA has been shown to possess potent anti-inflammatory properties. Inflammation is a common underlying cause of pain. AKBA is a pentacyclic terpenoid that is effective against a variety of inflammatory disorders such as chronic pain, cancer, osteoarthritis, etc. [19]. It inhibits the synthesis of pro-inflammatory mediators such as prostaglandins, leukotrienes, and cytokines by interfering with key enzymes and transcription factors involved in their production. The pharmacological properties of AKBA render them promising contenders for the management of masticatory myofascial pain. The anti-inflammatory, analgesic, and immunomodulatory effects that they possess have the potential to specifically target the inflammatory processes and pain pathways that are implicated in masticatory myofascial pain conditions. The capacity of AKBAs to suppress pro-inflammatory enzymes, such as 5-lipoxygenase (5-LOX) and cyclooxygenase-2 (COX-2), is considered to be the underlying mechanism for their anti-inflammatory effects. They also have the ability to hinder the activity of certain enzymes, thereby impeding the production of inflammatory mediators such as leukotrienes and prostaglandins. These mediators are known to contribute significantly to the initiation and sustenance of inflammatory processes. AKBAs also have the ability to modulate TRP channel activity, thereby exerting an influence on pain signaling and ultimately leading to a reduction in the perception of pain. Pain modulation is also influenced by the S adenosyl methionine. Boswellic acids have the ability to delay the activity of S-adenosylmethionine (SAMe) synthetase, which is the catalyst responsible for the transformation of methionine into SAMe, and has the ability to decrease SAMe production and subsequently influence subsequent methylation processes by inhibiting SAMe synthase. The alteration of SAMe levels has the potential to significantly impact diverse cellular processes, such as the metabolism and synthesis of neurotransmitters. AKBA has been widely used in chronic pain management in various disorders and demonstrated to have immunomodulatory, anti-inflammatory, cytotoxic, and antimicrobial effects.

COMT is an enzyme that controls the perception of pain within the body. It is involved in the breakdown and metabolism of neurotransmitters catecholamines such as dopamine, epinephrine, and norepinephrine. By adding a methyl group to these catecholamines, COMT renders them inactive and diminishes their effects. COMT is essential for modulating the levels of endogenous opioids, such as endorphins, in the brain and nervous system. Endogenous opioids are naturally occurring substances that alleviate pain. COMT is responsible for the degradation and inactivation of these opioids, which influences their availability and duration of action. Variations in the COMT gene can influence the activity and efficacy of the enzyme. The most studied genetic polymorphism is the Val158Met polymorphism, which results in a functional change in COMT activity. Individuals with the Met/Met genotype have lower COMT activity and higher levels of endogenous opioids, which may contribute to a greater pain tolerance. In contrast, individuals with the Val/Val genotype have higher COMT activity and lower levels of endogenous opioids, which may contribute to decreased pain tolerance. COMT's role in pain is not restricted to endogenous opioids but also to the methylation pathway where SAMe is the principal methyl donor to most methyltransferase enzymes The activity ofCOMT can also affect the levels of other neurotransmitters, such as dopamine, which are involved in pain modulation. Variations in COMT activity have been linked to differences in pain sensitivity, medication responses, and the development of chronic pain conditions [20].

Targeting COMT is complex and unpredictable. COMT is a central regulator of catecholamine levels in the pain perception pathway and is a crucial regulator of pain perception, cognitive function, and mood [21]. This study is based on a genomics-based drug design approach targeting COMT with the natural compound AKBA, which is the most bioactive ingredient of boswellic acids. This study aimed to create an in silico drug design for altering COMT in patients suffering from MMPS using AKBA as a target. With the newly discovered binding site on COMT, potential drugs can be developed using the natural compound. COMT gene polymorphisms are more common in 80% of the population with SNP in the COMT V158M gene. This polymorphism slows down the COMT pathway. The COMT pathway helps break down dopamine, norepinephrine, and epinephrine, which the body releases when under stress. Excessive dopamine/catecholamine causes over-stimulation of the frontal lobe, which slows down COMT activity which is the decreased activity of COMT enzyme that metabolizes catecholamine, causing chronic pain, anxiety, depression, Insomnia, etc. Over methylation is the critical cause of high catecholamines. This over-methylation drives the folic cycle too fast, producing excess BH4, and causing increased levels of adrenaline and serotonin, leading to increased pain symptoms. COMT is critical in deactivating neurotransmitters so that the quantity that binds to their receptors remains consistent and at physiologically acceptable levels. As previously stated, their behavior changes if these enzymes act too slowly or too quickly. COMT inhibits the activity of these neurotransmitters by inserting a methyl group into their structure, which is provided by SAMe [21].

SAMe is the principal methyl donor to most methyltransferase enzymes and COMT. SAMe can attenuate pain-related differential methylation by treating hypo-methylated and hyper-methylated genes when identifying dysregulated functions. Previous studies show a reversal of methylation in treating the frontal cortex with SAMe [22]. When the activity of catecholamine/dopamine in the frontal lobe is optimized and balanced, the slow activity of COMT can be treated. In a study, Gao et al. proved that AKBA can modulate SAMe as it directly interacts with methionine adenosyltransferase and inhibits its enzyme activity, decreasing the level of SAMe [23]. Hence the natural component AKBA has effects on SAMe which will be able to modulate COMT enzyme activity.

AKBA binding has a potential effect in enhancing the enzymatic activity ofCOMT which can be an effective drug for chronic pain sufferers as AKBA has the ability to modulate the SAMe which is the principal methyl donor of COMT which plays a major role in pain regulation due to excessive methylation. Due to the binding of AKBA on COMT, the ligand has the potential of reversing the methylation thus leading to decreased levels of SAMe which in turn can attenuate enhanced slow COMT activity. These findings provide new insights into the treatment of MMPS docking and molecular dynamic simulation results show that the stability and receptor-ligand interactions are very significant, with a docking score of -6.0 k/mol and the binding pocket was found relatively stable during the molecular dynamic simulations through RMSF for the residue surrounding the ligand in the protein complex [24].

The RMSD of AKBA was determined to be stable at 0.8-1.75 to 0-100 nanoseconds this was calculated using a protein-ligand complex system that is the average distance generated by the dislocation of a given atom. This proves that the stability of the AKBA ligand has an effective binding on COMT. On the basis of the present results, it can be interpreted that AKBA is a good lead compound that could enhance the enzymatic activity of COMT. However, preclinical and clinical studies are needed to investigate the in silico predictions in biological models for further therapeutic applications.

Limitations

The experimental validation of AKBA's efficacy in biological systems was not conducted in this study. Additionally, the impact of other genetic factors, pain mechanisms, and practical aspects such as dosage and bioavailability must be investigated. Future research should focus on confirming AKBA’s effects through experimental studies, exploring additional genetic variables, and evaluating the long-term safety and efficacy of AKBA as a treatment for MMPS.

## Conclusions

This study demonstrates the potential of AKBA, a natural compound derived from *Boswellia serrata*, to modulate COMT activity for the treatment of MMPS. The in silico analyses reveal stable interactions between AKBA and COMT, with promising implications for SAMe modulation and the management of pain. The molecular dynamics simulations and docking scores support the stability and effectiveness of AKBA's binding to COMT. However, these findings are preliminary, and further validation through rigorous in vitro and in vivo studies is essential. Future research should focus on confirming the therapeutic efficacy of AKBA, optimizing dosage and bioavailability, and assessing long-term safety and clinical applicability. Additionally, investigating the impact of genetic variations in COMT on treatment response will be crucial for personalized therapeutic strategies. Comprehensive preclinical and clinical trials are necessary to translate these insights into effective treatment modalities for MMPS.

## References

[1] Elbarbary M, Oren A, Goldberg M, Freeman BV, Mock D, Tenenbaum HC, Azarpazhooh A (2022). Masticatory myofascial pain syndrome: implications for endodontists. J Endod.

[2] Park HO, Ha JH, Jin MU, Kim YK, Kim SK (2012). Diagnostic challenges of nonodontogenic toothache. Restor Dent Endod.

[3] Costa YM, Ariji Y, Ferreira DM, Bonjardim LR, Conti PC, Ariji E, Svensson P (2018). Muscle hardness and masticatory myofascial pain: assessment and clinical relevance. J Oral Rehabil.

[4] Baker JS, Nolan PJ (2017). Effectiveness of botulinum toxin type A for the treatment of chronic masticatory myofascial pain: a case series. J Am Dent Assoc.

[5] Rao H, Maurya A, Kumar Raidas H (2024). In silico exploration of potential phytoconstituents from the bark extract of boswellia serrata for hemorrhoidal disease: molecular docking and molecular dynamics analysis. Chem Biodivers.

[6] Mladenovic I, Supic G, Kozomara R (2016). Genetic polymorphisms of catechol-o-methyltransferase: association with temporomandibular disorders and postoperative pain. J Oral Facial Pain Headache.

[7] Khawaja SN, Scrivani SJ (2019). Trigeminal autonomic cephalalgia and facial pain: a review and case presentation. J Oral Facial Pain Headache.

[8] Lin CH, Chaudhuri KR, Fan JY (2017). Depression and catechol-o-methyltransferase (COMT) genetic variants are associated with pain in parkinson’s disease. Sci Rep.

[9] Lim M, Nascimento TD, Kim DJ, Ellingrod VL, DaSilva AF (2021). Aberrant brain signal variability and COMT genotype in chronic TMD patients. J Dent Res.

[10] Braga SP, Fiamengui LM, da Silveira VR (2021). Insights for temporomandibular disorders management: from psychosocial factors to genetics - a case report. Spec Care Dentist.

[11] Siddiqui MZ (2011). Boswellia serrata, a potential antiinflammatory agent: an overview. Indian J Pharm Sci.

[12] Brendler T, Brinckmann JA, Schippmann U (2018). ustainable supply, a foundation for natural product development: the case of indian frankincense. J Ethnopharmacol.

[13] Jayasurya BR, Swathy J, Susha D, Sharma Sharma (2023). Molecular docking and investigation of boswellia serrata phytocompounds as cancer therapeutics to target growth factor receptors: an in silico approach. Int J App Pharm.

[14] Fatima SW, Alam S, Khare SK (2022). Molecular and structural insights of β-boswellic acid and glycyrrhizic acid as potent SARS-CoV-2 envelope protein inhibitors. Phytomed Plus.

[15] Caliebe RH, Scior T, Ammon HP (2021). Binding of boswellic acids to functional proteins of the SARS‐CoV‐2 virus: bioinformatic studies. Arch Pharm (Weinheim).

[16] Gao J, Cahill CM, Huang X (2018). S-adenosyl methionine and transmethylation pathways in neuropsychiatric diseases throughout life. Neurotherapeutics.

[17] Bron C, Dommerholt JD (2012). Etiology of myofascial trigger points. Curr Pain Headache Rep.

[18] Chang BT, Jiang HZ, Wei YJ (2022). Mangiferin: analgesic properties in neuropathic pain, molecular docking and meta-analysis. Phytomed Plus.

[19] Shin MR, Kim HY, Choi HY, Park KS, Choi HJ, Roh SS (2022). Boswellia serrata extract, 5-loxin®, prevents joint pain and cartilage degeneration in a rat model of osteoarthritis through inhibition of inflammatory responses and restoration of matrix homeostasis. Evid Based Complement Alternat Med.

[20] Brancher JA, Bertoli FM, Michels B (2021). Is catechol-O-methyltransferase gene associated with temporomandibular disorders? A systematic review and meta-analysis. Int J Paediatr Dent.

[21] Topham L, Gregoire S, Kang H (2021). The methyl donor S-adenosyl methionine reverses the DNA methylation signature of chronic neuropathic pain in mouse frontal cortex. Pain Rep.

[22] Morgese MG, Bove M, Francavilla M (2021). Sublingual AKBA exerts antidepressant effects in the aβ-treated mouse model. Biomol.

[23] Bai J, Gao Y, Chen L (2019). Identification of a natural inhibitor of methionine adenosyltransferase 2A regulating one-carbon metabolism in keratinocytes. EBioMedicine.

[24] Patel CN, Georrge JJ, Modi KM, Narechania MB, Patel DP, Gonzalez FJ, Pandya HA (2018). Pharmacophore-based virtual screening of catechol-o-methyltransferase (COMT) inhibitors to combat alzheimer's disease. J Biomol Struct Dyn.

